# The accuracy of the fullPIERS model in predicting adverse maternal and perinatal outcomes: evidence from a tertiary care maternity unit

**DOI:** 10.61622/rbgo/2025rbgo39

**Published:** 2025-07-15

**Authors:** Iury Gomes, Franz Marçal, João Victor de Carvalho Reis, Ana Paula dos Santos Silva, Lucas Sampaio, Leonardo Alves Moreira, Guilherme Lelis Costa, Mário Dias Correa, Zilma Silveira Nogueira Reis, Jussara Mayrink

**Affiliations:** 1 Universidade Federal de Minas Gerais Belo Horizonte MG Brazil Universidade Federal de Minas Gerais, Belo Horizonte, MG, Brazil.

**Keywords:** Pre-eclampsia, Maternal mortality, Infant, newborn, Pregnancy outcome, Developing countries

## Abstract

**Objective:**

Low- and middle-income countries face significant challenges in managing women diagnosed with pre-eclampsia, from making the clinical decision about whether to deliver to transferring these women to healthy facilities where they can receive appropriate care. The aim of this study was to evaluate the performance and accuracy of the fullPIERS model in a referral Brazilian maternity hospital - to assess maternal and fetal morbidity and impatient mortality at birth admission.

**Methods:**

A cross-sectional study analyzed pregnant women with preeclampsia diagnosis, between 2014 and 2023. The full PIERS model was applied to a database retrospectively collected and its accuracy to predict maternal and perinatal outcomes during the hospital stay was determined through a receiver operating curve.

**Results:**

Analyzing 207 pregnant women with fullPIERS had an Area Under the Curve (AUC) for adverse maternal outcome discrimination of 0.672 (0.576-0.767 95% CI, p<0.001) and AUC 0.582, (0.504-0.6661 95% CI, p = 0.041) for maternal and perinatal outcomes. Nevertheless, the model had no discrimination utility to assess perinatal outcomes (AUC 0.561, 0.480-0.642 95% CI, p = 0.642).

**Conclusion:**

The fullPIERS model had limited performance in identifying women at increased risk of adverse outcomes birth admission and absent utility to assess perinatal outcomes. Future studies, combining different tools and validated in low- and middle-income countries should be carried out to improve maternal health.

## Introduction

Preeclampsia affects approximately 5-10% of pregnant women worldwide.^(1)^ It is defined broadly as maternal hypertension after 20 weeks of gestation with signs or symptoms that indicate end-organ damage. Its prevalence may be influenced by many risk factors, such as previous history of preeclampsia, maternal age, obesity and the increase in the use of assisted reproduction techniques.^(2)^

Once preeclampsia is established, stroke, eclampsia, renal dysfunction, stillbirth, preterm delivery, and cerebral palsy are some of their maternal and fetal adverse consequences.^(3)^ These harmful consequences are correlated to the disease onset (before or after 34 weeks of gestation) and severity, but they are also correlated to health care facilities. For instance, in a previous report, generated from 2012 to 2014, the United Kingdom had only two maternal deaths due to hypertensive disorders. During the same period, Brazil recorded 971 maternal deaths.^(4)^ More than 90% of the maternal deaths related to hypertensive disorders occur in low- and middle-income countries (LMIC).^(5)^ Clinical care, health service organization and research priorities are some of the interventions that can explain the difference between HIC (high-income countries) and LMIC regarding maternal morbidity and mortality.^(4)^

In this context, it would be helpful if maternal and fetal adverse outcomes could be predicted in time to improve the care of a pregnant woman diagnosed with preeclampsia. This includes avoiding extreme premature interruption of gestation or transferring the woman to a health service with better conditions for mother and baby. The fullPIERS (Preeclampsia Integrated Estimate of Risk) model was developed with the aim of identifying the risk of fatal or life-threatening complications in women with preeclampsia within 48 h of hospital admission for the disorder.^(6)^ It is a free-of-charge risk calculator that includes gestational age data, maternal blood oxygen saturation, dyspnea or chest pain, hepatic transaminases, platelets and creatinine, which are routine laboratory parameters obtained from pregnant women with suspected preeclampsia. Although it is free of charge, its applicability worldwide, including low- and middle-income countries where it would be revolutionary, is modest. Therefore, our study aims to evaluate the performance and accuracy of the fullPIERS model in a hospital located in a scenario with shortage of resources and possibly to strengthen its utility in helping the reduction of maternal and fetal morbidity and mortality.

## Methods

**Figure 1:**
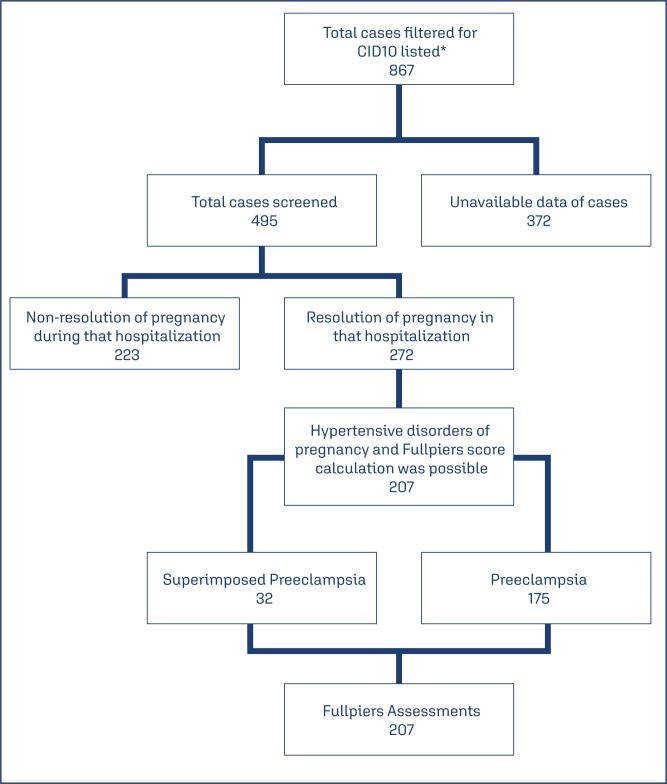
Flowchart of the analyzed pregnant women

This is a cross-sectional study involving pregnant women with the diagnosis of preeclampsia admitted at the maternity ward between 2014 and 2023 at the Hospital of Clinics at the Federal University of Minas Gerais, Brazil. This referral university hospital is a tertiary reference for high-risk pregnancies in the public health network, with ICU units for women and/or their newborn, performing approximately 1,500 deliveries annually. Pregnant women were not directly assessed; data was collected from their electronic medical records. The inclusion criteria were based on a list of the ICD-10 codes included as a diagnostic hypothesis in the discharge records of pregnant women admitted to the hospital between 2014 and 2023. The list is available as (Supplementary Material – Chart 1S) Of the 867 pregnant women initially selected, 372 were not available for analysis (due to lack of important records in the medical record or complete lack of access to the medical record). Of the remaining 495 medical records of pregnant women with diagnosis of preeclampsia analyzed, 272 accounted for women who gave birth during this hospitalization and were therefore included in the study. The distribution of the analyzed cases can be observed in the flowchart in figure 1.

In our study, we considered preeclampsia as the onset of hypertension after 20 weeks of gestation, followed by proteinuria. In the absence of it, the target organ dysfunction was considered to make the diagnosis according to *The 2021 International Society for the Study of Hypertension in Pregnancy classification, diagnosis & management recommendations for international practice* (Supplementary Material Chart 2S).^(1)^

Data was retrieved from electronic medical records. The variables included sociodemographic data, obstetric history, laboratory parameters, maternal and perinatal outcomes. The online fullPIERS calculator on the University of British Columbia website (https://pre-empt.obgyn.ubc.ca/home-page/past-projects/fullpiers/) was used to calculate the fullPIERS score for each case of pregnant women diagnosed with preeclampsia admitted to the study center. The parameters for fullPIERS were based on admission data. We tested the fullPIERS score to predict three composite adverse outcomes: maternal, perinatal, and conjoint maternal and perinatal outcomes. The choice to perform this approach was based on a previous publication from Guida et al.,^(7)^ which made a study with a similar pregnant population.

The adverse maternal outcomes considered in our analyses are as follows: eclampsia, HELLP syndrome, placental abruption, maternal hemorrhage, pulmonary edema, stroke or other central nervous system bleedings, renal or hepatic failure, and maternal death. The perinatal adverse outcomes considered were: fifth-minute Apgar score lower than 5 and stillbirth (intrauterine death). This study takes into account the same timeframe for the assessment of maternal outcomes as the original fullPIERS study, i.e. within 48 hours and seven days of admission.^(3,6)^

Descriptive analysis of the data obtained was performed, expressing frequencies, medians, and interquartile intervals among the participants. ROC (receiver operating characteristics) curves were built to analyze the performance of fullPIERS to predict adverse outcomes. Values of p<0.05 were considered statistically significant and an area under the curve (AUC) >0.7 indicated good discrimination, 0.5< AUC ≤0.7 indicated poor discrimination, and ≤ 0.5 was considered non-informative.^(8)^ The software used for analysis was SPSS Statistic version 26.

The Research Ethics Committee of the Federal University of Minas Gerais, Brazil (CEP-UFMG) approved the research project under the register number 6074180 (*Certificado de Apresentação de Apreciação Ética:* 67440323.8.0000.5149), according to the Resolution of the National Health Council 466/2012. Informed consent was waived due to the retrospective nature of the study.

## Results

We analyzed 207 pregnant women with preeclampsia, with a median age of thirty years old (IQR 12 years). Table 1 shows sociodemographic data, obstetrical history and maternal and perinatal outcomes of the population studied. The median gestational age at the diagnosis of preeclampsia was 35 weeks (IQR 5.43 weeks), with most cases diagnosed before 34 weeks (40.1%). Maternal and perinatal outcomes were evaluated within 48 hours and seven days from admission. Cesarean section was the mode of delivery in 156 pregnant women, with a frequency of 75.4%. Among the pregnant women with preeclampsia, 68.8% presented signs of severity and 57.5% received magnesium sulfate (119 pregnant women). HELLP syndrome complicated 20,3% of preeclampsia cases and placental abruption 5.3%. Eclampsia happened in six cases, all of them in hospitalized women. Table 1 shows that 59 of 207 women had an adverse maternal outcome within 48 hours and 7 days from admission. Stillbirth was observed in 14 cases (7.7%).

**Table 1:** Clinical and obstetrical characteristics of women with preeclampsia and superimposed preeclampsia included in the study

Variables		n(%)
Maternal Age, median IQR*		30(12)
Gestational age at onset of preeclampsia, median IQR*		35(5.43)
≥37 weeks		49(23.7)
34 – 36 weeks		75(36.2)
<34 weeks		83(40.1)
Route of delivery		
	Vaginal	51(24.6)
	Cesarean	156(75.4)
	Preeclampsia with severe features	141(68.8)
	Systolic blood pressure, median IQR*	150(20)
	Diastolic blood pressure, median IQR*	100(16)
	Use of antihypertensive drugs during pregnancy	87(42)
	Chronic hypertension superimposed preeclampsia	32(15.5)
	MgSO4 administration	119(57.5)
Maternal and perinatal outcomes		
	Eclampsia	6(2.9)
	HELLP syndrome	42(20.3)
	Placental abruption	11(5.3)
	Maternal death	0(0)
	5th minute Apgar score < 7 OR NICU** admission	72(34.8)
Stillbirth		14(7.7)

* IQR - (interquartile interval p25 – p75);

** NICU - Neonatal intensive care unit

Only 9 (4.33%) pregnant women had recorded information about chest pain or dyspnea. Table 2 shows the fullPIERS parameters assessed to determine the score.

**Table 2:** Assessment of clinical and laboratorial parameters of the fullPIERS score

Variable	Values
Presence of dyspnea or chest pain	9(4.33)
Oxygen saturation median IQR*	98(1)
Aspartate transaminase (U/L), median IQR*	23(15.25)
Alanine transaminase (U/L), median IQR*	21(17)
Platelets count (units/mm3), median IQR*	211(93)
Serum creatinine (mg/dL), median IQR*	0.6(0.23)

* IQR - (interquartile interval p25 – p75)

**Figure 2:**
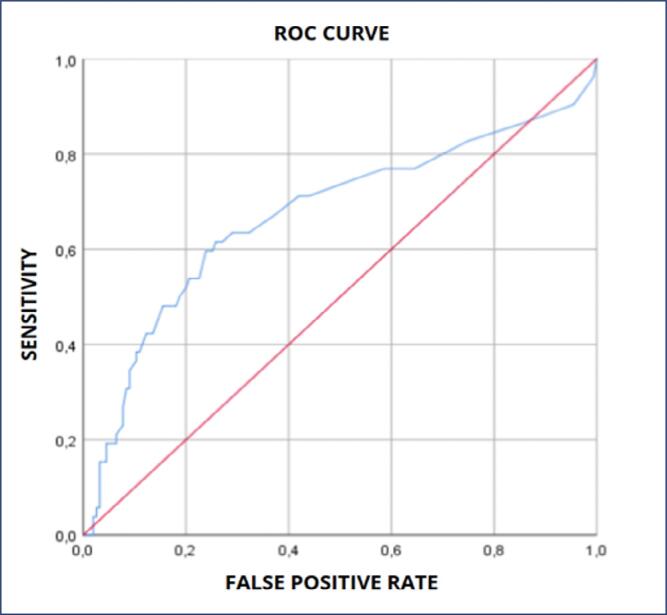
FullPIER to predict maternal outcomes

**Figure 3:**
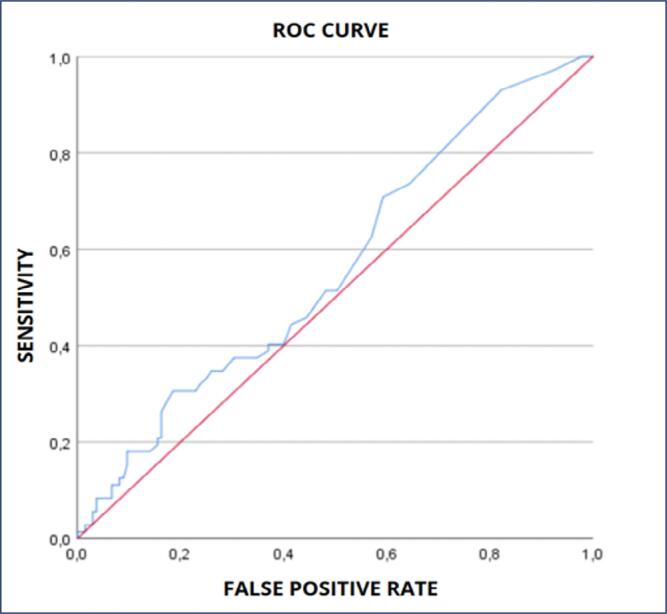
Full PIER to predict perinatal outcomes

**Figure 4:**
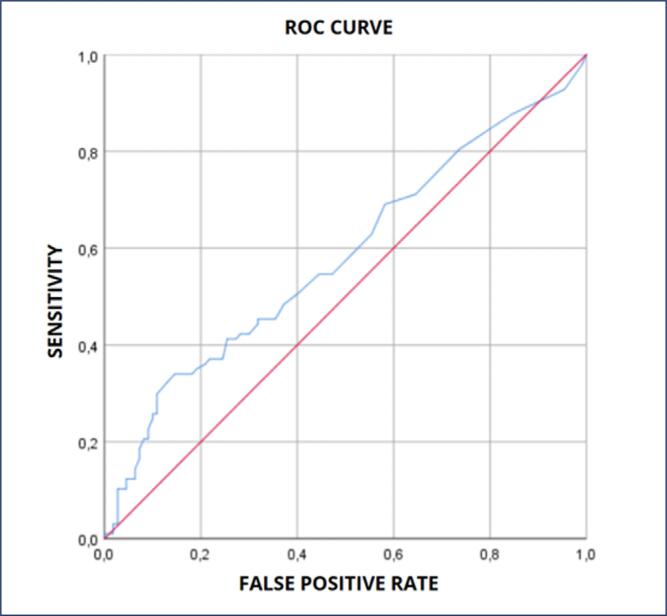
Full PIER to predict maternal and perinatal outcomes combined

The accuracy of the fullPIER to predict maternal outcomes showed poor discrimination (AUC = 0.672 (0.576-0.767 95% CI, p<0.001) (Figure 2). For a sensitivity of 65,4% and a specificity of 65,2%, the cut- off value of fullPIERS was 1.05. When analyzed to predict perinatal outcomes, the model had no discrimination utility to assess perinatal outcomes (AUC = 0.561, 0.480-0.642 95% CI, p = 0.642) (Figure 3). Taking maternal and perinatal outcomes together, a total of 97 adverse outcomes took place, performing 46.9% of the total of pregnant women. The accuracy of fullPIERS to predict those outcomes together demonstrated poor discrimination (AUC 0.582, 0.504-0.6661 95% CI, p = 0.041) (Figure 4).

## Discussion

This study showed that the performance of the fullPIERS model to distinguish between pregnant women with potential severity from those with favorable outcomes in our population had low accuracy. Among women with preeclampsia, 68.1% had severe features and 20.3% had the diagnosis of HELLP syndrome. Nearly 16% of the women who were supposed to receive magnesium sulfate were not receiving it. Taken together, 46.9% of the pregnant women with preeclampsia analyzed presented a maternal or a perinatal adverse outcome.

Originally, the fullPIERS model was developed and internally validated in a cohort study involving high-income countries: Canada, New Zealand, Australia, and the United Kingdom. The original PIERS study by von Dadelszen et al.^(9)^ demonstrated that the algorithm was able to predict adverse maternal outcomes within 48 h of admission, with an AUC of 0·88 (95%CI 0·84–0·92). Since then, the model has been validated in different settings, seen as a tool to maximize the temporal expectant management of pregnancy and a way to optimize pregnancy care. The performance varies according to those settings. Great predictive performance was shown in a study in the Netherlands, with an AUC of 0·97 (95% CI 0·94–0·99).^(10)^ This cohort is similar to the cohort where the internal validation of the model fullPIERS occurred. On the other hand, studies conducted in some low- and middle-income countries showed modest performance of the fullPIERS model to predict perinatal outcomes. Ukah et al.^(3)^ showed an AUC of 0.77 in a population of 5 LMIC (the population of the mini-PIERS validation): Brazil, Fiji, Uganda, South Africa and Pakistan.

Two Brazilian studies showed a better predictive ability of the fullPIERS score. The study by Guida et al.^(7)^ showed an AUC of 0.845, despite being conducted in a hospital of similar complexity. Compared with the study by Almeida et al.,^(11)^ the current study evaluated fullPIERS in a similar context but with different approaches: our study of 207 pregnant women showed limited utility of the model (AUC 0.672 for maternal outcomes), whereas Almeida's study of 325 women found an AUC of 0.72 for adverse maternal outcomes.^(11)^

We hypothesize that a possible reasoning for the fullPIERS model low prediction performance in our study (and perhaps in other ones in LMIC) is the presence of more comorbidities in our population, less availability of resources and differences in disease management, with less expectant management of preeclampsia. We supposed that: LMIC might be more likely to have shorter intervals between admission and delivery and more co intervention in pregnant women with preeclampsia. Thus, the natural history of preeclampsia was truncated by expedited delivery.

Our sample had a high rate of C-section of 75.4%. Considering the high rate of early-onset preeclampsia and HELLP syndrome in our population, probably, they would have contributed to this high rate of C-section delivery, in accordance with the institutional protocol. However, Brazil has demonstrated over the years an increment in C-section rates, with rates of 42.9% in national public healthcare and 87.9% within private healthcare, as demonstrated by Nakamura-Pereira et al.^(12)^ Unfortunately, these numbers seem to follow a global trend.^(13,14)^

Preeclampsia with signs of severity was diagnosed in 141 participants (68.8%). The HELLP syndrome complicated 20.3% of pregnancies analyzed. Surprisingly, almost 16% of the women who were supposed to receive magnesium sulfate did not receive it. The magnesium sulfate is well established as the medication to halve the risk of eclampsia and maternal death, since the beginning of this century.^(15)^ Hence, one could find it quite surprising that in a tertiary hospital one in five women who are supposed to receive magnesium sulfate did not receive it and is exposed to unnecessary risks. Fear of toxicity and misinformation are probably among the reasons for this discouraging reality. Our study showed 3% of eclampsia, which is the main cause of death related to hypertension in Brazil.^(16)^ Six pregnant women had seizure crises in hospital, after admission and under the care of the health team. Given the underuse of magnesium sulfate, these six cases could have been avoided by the timely use of the medication designed to prevent eclampsia.

Considering maternal and perinatal adverse outcomes, 46.9% of the participants presented an unfavorable outcome. This number reinforces the severity of preeclampsia and the necessity to abandon concepts like "mild" preeclampsia, already proposed since 2014, given its great potential for complications.^(17)^

Dyspnea and chest pain were rarely mentioned in the medical records. Unfortunately, this data cannot be checked and a possible reason for the lack of information is the fact that this is not routinely inquired by the healthy team to the patients. These are important symptoms that should be incorporated at least to the first interview of pregnant women with preeclampsia diagnosis.

Recently, the interest in improving the fullPIERS model performance with maternal seric biomarkers, especially sFlt-1 (soluble FMS-like tyrosine kinase-1) and PlGF (placental growth factor) ratio regarding their property of adverse maternal prediction has increased.^(18)^ In a study from Serbia involving preeclampsia cases with early-onset diagnosis, the accuracy (AUC) of sFlt-1/PlGF ratio was 0.853 and the accuracy of fullPIERS was 0.628.^(18)^ However, the cost-effectiveness of this association in LMIC settings still needs to be studied. Even though the fullPIERS model applies information routinely available at presentation with preeclampsia in general, its applicability especially in primary care settings of LMIC still faces barriers. The inclusion of laboratory tests like sFlt-1 and PlGF (sometimes not performed in those settings) and external validation limitations make this tool underutilized.

More recently, the same group that developed the fullPIERS model proposed a machine learning model with the same purpose as the first, but developed and validated in high-, low- and middle-income countries. This time, instead of logistic regression, they developed and internally validated an 18-variable model for maternal risk stratification using a machine learning approach.^(18)^ The population included 11 low-, middle- and high-income countries, including sub-Saharan Africa, South America, South Asia and Oceania, with a total of 8843 participants. The model identified almost 40% of women with preeclampsia for whom changes in care should be made: place of care, transfer of care, antenatal and postnatal monitoring, co-interventions and timed birth. The performance appears to be better than that demonstrated in the external validation of the fullPIERS model in lowand middle-income countries, with an AUC of 0.8, compared with 0.68 in the original study.^(3)^ Of course, this needs to be tested in future studies.

Our study seems to have several limitations, and outcomes deserve caution in generalization. The main limitation of this study is the limited power due to the small sample size and a high proportion of missing cases of preeclampsia, in view of database changing in the local hospital, and the difficulty to access medical recorders. For this reason, a selection bias should be considered. Due to the small sample size, it wasn't possible to do a subgroup analysis of pregnant women with a gestational age of less than 34 weeks. This subgroup is actually the one of greatest interest, because after this gestational age, cases of preeclampsia with signs of severity benefit from bringing the birth forward.^(19,20)^

On the other hand, this study also has strengths. The conduction of this study in a low resourced setting is particularly useful, considering the high prevalence of morbidity and mortality attributable to preeclampsia in LMIC.

## Conclusion

In our study, the performance of the fullPIERS model in predicting maternal and perinatal adverse outcomes was poor compared with other cohorts. We speculate that the heterogeneity of the population, the high proportion of morbidity and the practice of expedited delivery may have influenced our results. Future studies with larger sample sizes are needed to confirm our findings. We also encourage future studies using machine learning models with a higher number of variables and perhaps a powerful approach validated in LMIC, which may help in the management of preeclampsia.

## Supplementary Material

**Chart 1S:** List of CIDs included in the survey

CID-O10 (0 to 9) - Pre-existing hypertension with superimposed pre-eclampsia
CID-O14 (0 to 9) - Pre-eclampsia
CID-O12 (0 to 9) - Gestational hypertension (induced by pregnancy) without significant proteinuria
CID-O15 (0 to 9) - Eclampsia
CID-O16 (0 to 9) - Unspecified hypertension during pregnancy, childbirth, and the puerperium
Z35 - Supervision of high-risk pregnancy
P35 - Congenital viral diseases
O36.4 - Maternal care for suspected fetal distress.

**Chart 2S:** Diagnostic criteria for preeclampsia

Systolic blood pressure ≥140 mmHg and/or diastolic blood pressure ≥90 mmHg on at least 2 occasions at least 4 hours apart after 20 weeks of gestation in a previously normotensive patient AND the new onset of 1 or more of the following*:
Proteinuria ≥0.3 g in a 24-hour urine specimen or protein/creatinine ratio ≥0.3 (30 mg/mmol) in a random urine specimen or dipstick ≥2+ if a quantitative measurement is unavailable
Platelet count <100,000/microL
Serum creatinine >1.1 mg/dL (97.2 micromol/L) or doubling of the creatinine concentration in the absence of other kidney disease
Liver transaminases at least twice the upper limit of the normal concentrations for the local laboratory
Pulmonary edema
New-onset and persistent headache not accounted for by alternative diagnoses and not responding to usual doses of analgesics
Visual symptoms (eg, blurred vision, flashing lights or sparks, scotomata)

**Source:** Adapted from Magee et al. (2022).^(1)^
